# A model-based cost-effectiveness analysis of fracture liaison services in China

**DOI:** 10.1007/s11657-022-01170-1

**Published:** 2022-10-05

**Authors:** Nannan Li, Lei Si, Annelies Boonen, Joop P. van den Bergh, Mickaël Hiligsmann

**Affiliations:** 1 Department of Health Services Research, CAPHRI Care and Public Health Research Institute, Maastricht University, P.O. Box 616, 6200 MD Maastricht, The Netherlands; 2 The George Institute for Global Health, UNSW Sydney, Kensington, Australia; 3 School of Health Sciences, Western Sydney University, Campbelltown, Australia; 4 Department of Internal Medicine, Division of Rheumatology, Maastricht University Medical Centre, and CAPHRI Research Institute, Maastricht University, Maastricht, The Netherlands; 5 Department of Internal Medicine, VieCuri, Medical Centre, Venlo, The Netherlands; 6 Department of Internal Medicine and NUTRIM Research Institute, Maastricht University Medical Centre, Maastricht, The Netherlands

**Keywords:** Cost-effectiveness, Fracture liaison services, Fracture, Osteoporosis

## Abstract

***Summary*:**

This study is a model-based cost-effectiveness analysis of fracture liaison services (FLS) in China, suggesting that FLS could potentially lead to lifetime cost-saving in patients who have experienced a fracture. However, Chinese-specific real-world data is needed to confirm the results of our study.

**Purpose:**

The study aimed to assess the potential cost-effectiveness of fracture liaison services (FLS) from the Chinese healthcare perspective with a lifetime horizon.

**Methods:**

A previously validated Markov microsimulation model was adapted to estimate the cost-effectiveness of FLS compared to no-FLS. The evaluation was conducted in patients aged 65 years with a recent fracture. Treatment pathways were differentiated by gender, FLS attendance, osteoporosis diagnosis, treatment initiation, and adherence. Given the uncertainty in FLS cost, the cost in the base-case analysis was assumed at US$200. Analyses were also performed to determine the maximum cost for making the FLS cost-saving and cost-effective at the Chinese willingness-to-pay (WTP) threshold. One-way sensitivity analyses were conducted.

**Results:**

When compared with no-FLS, the FLS was dominant (lower costs, higher quality-adjusted life years) in our target population at the FLS cost of US$200 per patient. For every 100 patients who were admitted to the FLS, approximately four hip fractures, nine clinical vertebral fractures, and three wrist fractures would be avoided over their lifetimes. Our findings were robust to numerous one-way sensitivity analyses; however, the FLS was not cost-effective in patients aged 80 years and older.

**Conclusion:**

FLS could potentially lead to lifetime cost-saving in patients who have experienced a fracture. Our study informs the potential cost-effectiveness of FLS and the knowledge gap in China; more future research incorporating Chinese-specific real-world data are needed to confirm the results of our study and to better evaluate the cost-effectiveness of FLS in China.

**Supplementary Information:**

The online version contains supplementary material available at 10.1007/s11657-022-01170-1.

## Introduction

Osteoporosis causes loss of bone mass and deterioration of bone microarchitecture, which is the main risk factor for fragility fractures. Osteoporosis-related fractures can lead to an increased risk of subsequent fractures and reduced quality of life. In the context of the aging population and increasing life expectancy, osteoporosis places a large medical and economic burden on healthcare systems [1]. This burden is more profound in countries like China, which is stressed by limited healthcare resources and a large population [2]. The estimated age-standardized lifetime prevalence of osteoporosis was 6.46% and 29.13% for Chinese men and women aged 50 years and older, respectively [3]. In one study performed in eight provinces of China [4], the estimated osteoporosis-related fracture incidence rate was 160.3/100,000 person-years, with 120.0 and 213.1/100,000 person-years in men and women aged 50 years or older, respectively. The annual cost of hospitalization was estimated in a recent Chinese study [5], ranging from US$3142 for hand and wrist fractures to US$10,355 for hip fractures per patient.

Despite the availability of various effective pharmaceutical interventions for fracture prevention, osteoporotic fractures are still undertreated [6]. One study [7] explored the management of osteoporosis after a fragility fracture among postmenopausal women in six Asian countries, reporting a substantial treatment gap (67%) 6 months after the index fracture. The gaps were even more profound in mainland China, where the treatment initiation rate was lower than the average in these six Asian countries. Another Chinese study (in which the diagnosis rate of osteoporosis was 56.8%) reported that a bone mineral density (BMD) measurement had never been conducted in 42% of patients with fragility fractures, that nearly 30% of patients had never received basic calcium and/or vitamin D supplementation, and that following fragility fractures, only 28% of elderly patients were prescribed with pharmaceutical treatment for osteoporosis besides calcium and vitamin D [8].

In response to the care gap in the elderly after fragility fractures, the International Osteoporosis Foundation (IOF) launched the Capture the Fracture (CTF) Campaign in 2012 to facilitate implementation of the Post-Fracture Care (PFC) coordination program, such as fracture liaison services (FLS), for secondary fracture prevention. FLS is advocated as the best practice covering all aspects, including patient identification, education, risk evaluation, treatment, and long-term monitoring, to directly improve patient care and reduce spiraling fracture-related healthcare costs. A recently published meta-analysis indicated that FLS reduced the risk of subsequent fractures by 30% [9]. To date (13 June, 2022), 739 FLS (registered in the CTF Campaign) have been implemented in 50 countries worldwide. In recent years, the number of FLS in the Asia–Pacific (AP) region has risen rapidly [10], with 41 FLS in China currently registered in the CTF Campaign (mainland China: 6; Taiwan: 31; Hong Kong: 4). However, in comparison with European countries, the number of FLS remains limited in China, and the intensity of implementing FLS is inadequate.

To help the implementation of FLS, it is important to assess the cost-effectiveness of FLS models. Given limited healthcare resources and budgets, economic evaluations are used increasingly nowadays to support the setting of priorities in healthcare. Accordingly, in recent years, several cost-effectiveness analyses of FLS have been conducted, and 16 studies published up to December 2016 were summarized in a systematic review by Wu et al. [11]. This review suggested that FLS were cost-effective compared with usual care or no treatment, regardless of the program intensity or the country; 47% of studies even documented cost-savings. However, economic evidence regarding the FLS implementation in China is largely lacking, and due to the limited transferability of cost-effectiveness analyses between countries, it is important to investigate the potential economic value of FLS from the Chinese healthcare perspective with a lifetime horizon. The objective of this study was therefore to assess the potential cost-effectiveness of FLS in China. Given the uncertainty in FLS costs, analyses were also performed to determine the maximum cost for making the FLS cost-saving and cost-effective at the Chinese willingness-to-pay (WTP) threshold.

## Methods

A previously validated Markov microsimulation model [12] was adapted to estimate the cost-effectiveness of FLS compared to no-FLS with a lifetime horizon from the Chinese healthcare perspective. The individual-level simulation allows the tracking of patient characteristics and disease histories and avoids unnecessary transition restrictions [13]. In this way, the number and the type of subsequent fractures were recorded for each individual using “tracker variables.” The model was developed using TreeAge Pro 2021 software (TreeAge Pro Inc., Williamston, MA, USA) and was conducted in line with recommendations for the conduct of economic evaluations in osteoporosis provided by the European Society for Clinical and Economic Aspects of Osteoporosis, Osteoarthritis and Musculoskeletal Diseases (ESCEO) and the US branch of the International Osteoporosis Foundation (IOF) [14] and with the Consolidated Health Economic Evaluation Reporting Standards 2022 (CHEERS 2022) Statement [15]. Appendices I and II include details of the two checklists. A description of the model is provided here below.

### Model structure

The population of our analysis was patients who had recently suffered a fracture; both males and females were included because of large differences in the probability of osteoporosis, fracture incidence, and risk of subsequent fractures. The prevalence of osteoporosis was derived from the study of Wang et al. [16]; osteoporosis was defined as individuals with BMD T scores of − 2.5 or less in any sites (lumbar spine L1 to L4, femoral neck, or total hip). The base-case population had a starting age of 65 years old, which was aligned with the mean age of most FLS studies summarized in a systematic review on the effectiveness of FLS [9].

**Fig. 1:**
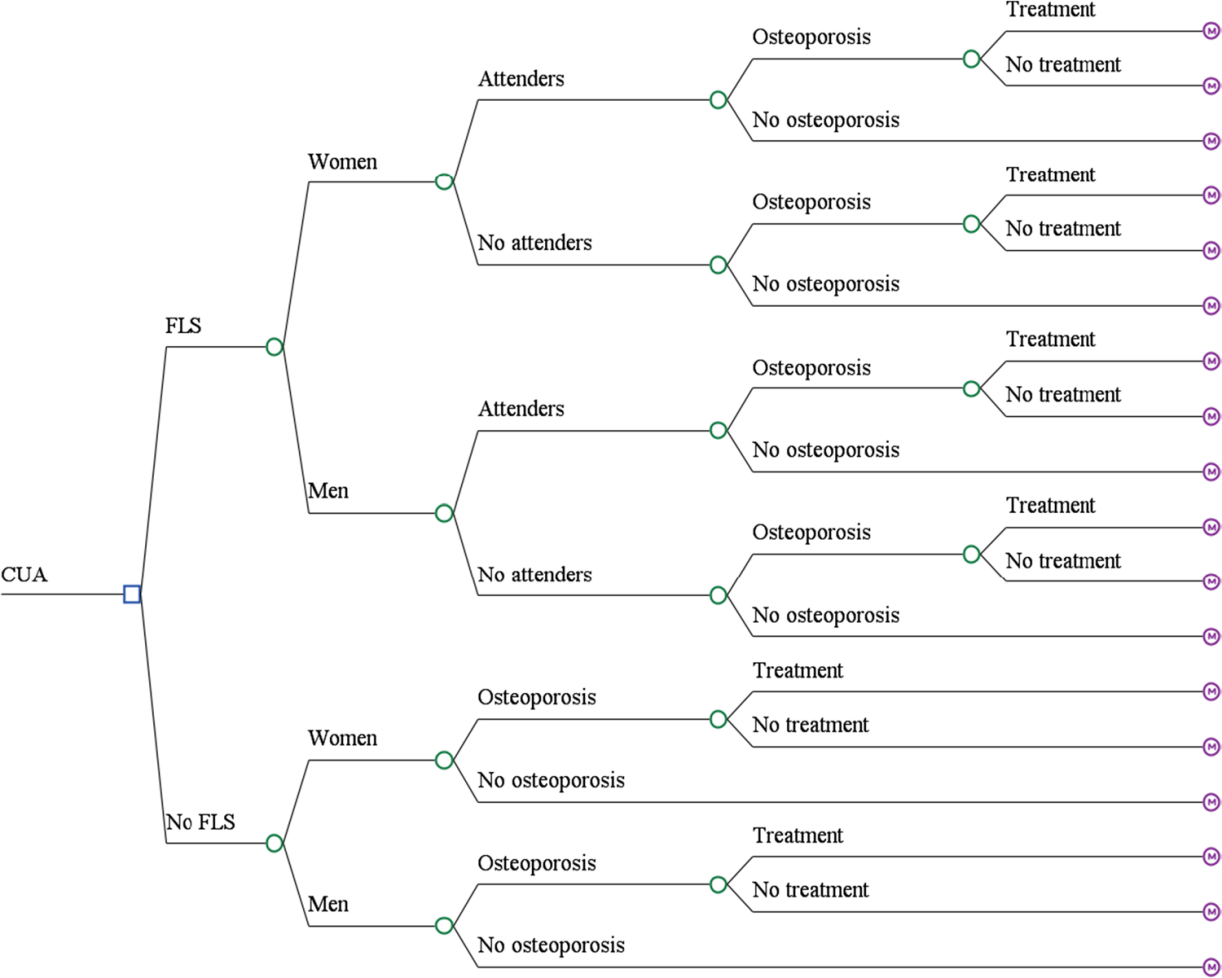
Patient pathways for FLS and no-FLS group (CUA, cost-utility analysis; FLS, fracture liaison services)

As displayed in Fig. 1, the economic model consisted of a decision tree (to determine the treatment pathway), followed by a Markov model. Treatment pathways were differentiated by gender (male/female), attenders/non-attenders, diagnosis of osteoporosis or not, and treatment initiation or not, leading to a total of 18 possible pathways.

**Fig. 2:**
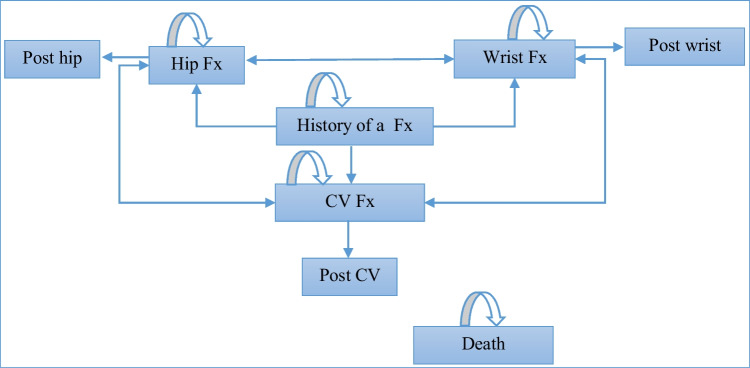
Structure of the Markov model (Fx, fracture; CV Fx, clinical vertebral fracture)

After each pathway, patients entered a Markov model (see Fig. 2), where all patients started in the health state of “a recent fracture” and could transit between future fracture health states (hip, vertebral, and wrist), their corresponding post-fracture states, and death. Patients could experience multiple fractures at the same site or multiple sites. If a patient died, he/she would remain in the “death” state for the rest of the simulation. In line with ESCEO-IOF guideline [14], the cycle length of this model is 6 months; each patient would be followed until they died or reached the age of 100 years.

The primary outcome was the incremental cost-effectiveness ratio (ICER) between FLS and no-FLS care, expressed as incremental cost per quality-adjusted life year (QALY) gained. The discount rate of 5% was used for both costs and QALYs as recommended by the China Guidelines for Pharmacoeconomic Evaluations (2020 edition) [17]. Data used for the model are shown in Table 1.

**Table 1:** Key parameters derived from literature for base-case analysis

Parameter	Female	Male	Data source
Gender (%)	57.7	42.3	[16]
Proportion of osteoporosis (%)	37.1 (60–69 years), 51.3 (70–79 years), 67.5 (80 + years)	5.4 (60–69 years), 12.3 (70–79 years), 21.9 (80 + years)	[16]
Fracture risk and mortality			
Fracture incidence (annual rate per 1000 person-years)			
Hip	0.96 (65–69 years), 2.34 (70–74 years), 4.08 (75–79 years), 6.44 (80–84 years), 6.59 (85–89 years), 8.67 (90 + years)	0.65 (65–69 years), 1.26 (70–74 years), 2.37 (75–79 years), 5.19 (80–84 years), 5.71 (85–89 years), 8.35 (90 + years)	[18]
Vertebral	5.64 (65–69 years), 8.74 (70–74 years), 12.05 (75–79 years), 21.19 (80–84 years), 26.89 (85 + years)	0.95 (65–69 years), 2.26 (70–74 years), 4.50 (75–79 years), 5.94 (80–84 years), 9.54 (85 + years)	[19]
Wrist	12.95 (65–69 years), 13.17 (70–74 years), 13.87 (75–79 years), 15.01 (80–84 years), 15.10 (85–89 years), 13.97 (90 + years)	3.27 (65–69 years), 2.79 (70–74 years), 3.13 (75–79 years), 4.75 (80–84 years), 4.78 (85–89 years), 3.23 (90 + years)	[20]
Relative risk of having a subsequent fracture	1.95	3.47	[21]
Relative risk of fracture for individuals with osteoporosis			
Hip	3.91 (65–69 years), 3.31 (70–74 years), 2.6 (75–79 years), 2.04 (80–84 years), 1.92 (85 + years)		[17, 22]
Vertebral	2.59 (65–69 years), 2.15 (70–79 years), 1.82 (80 + years)		[22, 23]
Wrist	1.78 (65–69 years), 1.60 (70–79 years), 1.45 (80 + years)		[22, 24, 25]
All-cause mortality (per 1000) for the general population	13.06 (65–69 years), 24.36 (70–74 years), 40.89 (75–79 years), 73.98 (80–84 years), 115.29 (85–89 years), 180.24 (90–94 years), 219.46 (95–99 years), 436.34 (100 + years)	21.26 (65–69 years), 37.02 (70–74 years), 59.13 (75–79 years), 98.56 (80–84 years), 146.53 (85–89 years), 211.66 (90–94 years), 212.07 (95–99 years), 507.28 (100 + years)	(https://www.chinayearbooks.com/tags/china-statistical-yearbook)
Excess mortality after a subsequent fracture	1.91	2.99	[26]
Direct fracture cost (US$2020)			
Hip, first 1 year	11,166		[27]
Hip, long-term annually	4799		[28]
CV, first 1 year	6328		[27]
Wrist, first 1 year	2162		[5]
Health state utility values			
Baseline (patients with a fracture)	0.7		[29]
Hip (1st year/subs. years)	0.55/0.86		
CV (1st year/subs. years)	0.68/0.85		
Wrist (1st year/subs. years)	0.83/0.99		
Treatment			
Treatment effects of oral alendronate (relative risk of fracture)			
Hip	0.67		[30]
Vertebral	0.45		
Wrist	0.81		
Treatment cost (USD 2020)			
Drug cost (6 months)	392.31		[31]
DXA cost	87.57		
GP cost per person	10.3		
Side effect cost	0.041 extra GP consultations during the first cycle (6 months) and 0.021 GP consultations during the following cycles		[32]
Treatment persistence (%)	56 (6 months), 50 (1 year), 33 (2 years), 21 (3 years), 12 (4 years), 6 (5 years)		[33, 34]
FLS-related data			
Treatment initiation (%)	FLS pathway: 38 no-FLS pathway: 17.2		[35]
Treatment adherence (%)	FLS pathway: 57 no-FLS pathway: 34.1		[35]
One-off FLS cost (USD 2020)	200 (base case)		Assumption based on three studies [36–38]
FLS attendance rate (%)	66		[9]

*CV* clinical vertebral fracture, *DXA* dual-energy X-ray absorptiometry, *FLS* fracture liaison service, *GP* general practice

### Treatment pathways

The FLS pathway was differentiated from the no-FLS pathway mainly in terms of the proportion of patients receiving actual FLS care (i.e., incurring FLS costs and having a higher likelihood of starting anti-osteoporosis medication), treatment adherence, and the presence of FLS costs. For both FLS and no-FLS pathways, we assumed that 57.7% of patients were females. According to a recent Chinese study that summarized the prevalence of clinical fracture in the past 5 years [16], the proportion was comparable to a recently published study (51.29% of patients were females) which included 39,300 patients aged over 45 years with a fracture in Jiangsu, China [5]. To make the FLS and no-FLS pathways comparable, the same (age and gender-specific) proportion of patients having osteoporosis was assumed. Afterward, in both FLS and no-FLS, patients entered different branches in terms of their treatment status (no osteoporosis, osteoporosis + no treatment, osteoporosis + treatment). In our model, we made a conservative assumption that patients without osteoporosis did not initiate treatment (although some local guidelines suggest that patients with grade 2 or 3 vertebral fractures should initiate treatment irrespective of their BMD status, the relevant data was lacking in China). For patients diagnosed with osteoporosis, some patients would initiate treatment, and the difference between the FLS and no-FLS pathways was that a higher proportion of patients in the FLS pathway initiated treatment compared to patients in the no-FLS pathway (i.e., 38% for FLS vs. 17.2% for no-FLS), according to a systematic review and meta-analysis [23]. In addition, the treatment adherence in the FLS pathway was also higher given the positive role of the FLS coordinator who usually provided treatment advice and long-term monitoring for patients in the FLS.

Moreover, in the FLS pathway, patients were further divided into attenders and non-attenders. The proportion of patients who attend the FLS was defined as the number of patients actually attending the FLS divided by the total number of patients eligible or invited for the FLS (and thus assuming all patients with fractures are invited). FLS attendance means that the full assessment (laboratory test), including advice on treatment, has been executed. Based on two previous literature reviews [9, 23], the average FLS attendance rate was estimated at 66% and used in our study. We further assumed that attenders and non-attenders have the same baseline fracture risk.

### Osteoporosis prevalence, fracture risk, and mortality

Given the lack of osteoporosis prevalence data for patients with a recent fracture in China, age- and gender-stratified osteoporosis prevalence rates for the Chinese general population were used to determine the initial probability of the simulated subjects being osteoporotic [16], for both attenders and non-attenders. The proportion of 65-year-old female and male patients having osteoporosis was 37.1% and 5.4%, respectively. Considering that the prevalence of osteoporosis in the fractured population might be higher than in the general population, the baseline prevalence of osteoporosis was increased by 20% and 40% separately in one-way sensitivity analyses.

The gender-specific annual incidence rates of hip and vertebral fracture in the general population were derived from the Hefei osteoporosis project [18] and the epidemiological study of Hong Kong [19], respectively. In the absence of estimates of the annual incidence rate of wrist fracture in the Chinese population, a Norwegian study [20] was used, multiplying by 0.72 to adjust for the Asian population, as indicated in this article. Rates were converted to risk. In addition, considering our patients had a fracture at baseline, the increased risk of having a subsequent fracture was assumed (relative risk (RR) was 1.95, 3.47 for females and males, respectively), which was taken from the Dubbo Osteoporosis Epidemiology Study (DOES) [21]. However, given no relevant high-quality data in China on the increased risk following a second, third subsequent fracture, etc., we therefore conservatively did not assume, during simulation, the extra increased risk for the occurrence of new fractures.

As patients with osteoporosis have an increased risk of fracture in comparison with those without osteoporosis, the initial probabilities (we mentioned above) were then adjusted to reflect the fracture risk of patients with osteoporosis. The RR was extracted from a recently published cost-effectiveness analysis [39] which estimated the age-stratified RR based on previous studies [24, 25] using previously validated methods [22]. Of note, given the lack of RR data for patients aged 60–64 years (for sensitivity analysis purposes), we assumed the same RR as patients aged 65 years. In addition, considering that not all fractures were attributable to osteoporosis, the age- and gender-specific osteoporosis attribution probability [40] was applied to make the further adjustment.

Baseline mortality rates for the age- and gender-stratified Chinese population were obtained from the China Public Health Statistical Yearbook. An increased mortality risk after hip fracture and clinical vertebral fracture was assumed for both genders [26], which is in line with previous economic studies [41]. Given that comorbidities could also be a contributing factor for excess mortality, we further took into account that only 25% of the excess mortality following fractures was attributed to the fractures themselves [42, 43].

### Fracture cost

A healthcare perspective was used for cost estimation. Costs of hip and vertebral fractures referred to hospitalization costs deriving from a recently published Chinese study [5]. As this study classified wrist and hand fracture as one category, the cost of wrist fracture was obtained from another Chinese study [27]. In addition, hip fractures are also associated with long-term costs. The probability of admission to a nursing home after a hip fracture is usually very low in China and was assumed to be 5%, based on expert opinion. The annual costs for nursing home residence were retrieved from a previous study [28] which was based on prices recommended by the Chinese government. All costs were converted to the 2020 US dollar in the analysis.

### Utility values

The baseline utility value (0.70) for patients with a history of fracture was estimated based on 12-month utility data after a fracture of the International Costs and Utilities Related to Osteoporotic Fractures Study (ICUROS) [29]. This study assessed the quality of life of patients with fractures from 11 countries including 2808 patients. The health state utility values (HSUVs) for the first and subsequent years after a fracture were calculated using a multiplicative approach. The fracture-specific multipliers were also obtained from the ICUROS study [29].

### Treatment effects

Oral bisphosphonates are commonly used as the first-line therapy for osteoporosis management in China [44]. In this study, we therefore assumed that patients initiated treatment with weekly oral alendronate. The pooled efficacy data for bisphosphonates of the National Institute for Clinical Health and Excellence (NICE) was applied [30]. This study suggests that oral bisphosphonates resulted in a relative risk (RR) of 0.67, 0.45, and 0.81 for hip, vertebral, and wrist fracture, respectively. The treatment duration was 5 years maximum (which was consistent with Chinese guidelines for diagnosis and treatment of osteoporosis) [44]. After stopping medication, it was assumed a linear decrease of the effects for a duration similar to the duration of therapy, in line with previous economic analyses of oral bisphosphonates [45] and clinical data [46].

The real-world persistence data for weekly bisphosphonates was obtained from a Japanese study [33]; persistence refers to the duration of time from initiation to discontinuation of the therapy, which was based on prescription data in 13 university hospitals in Japan, showing that the cumulative persistence rates with weekly bisphosphonates were 50%, 33%, 21%, 12%, and 6% at the end of first, second, third, fourth, and fifth years, respectively. The persistence rate for the first 6 months (56%) was estimated according to the study of Chandran et al. [34]; the same ratio between the 6 and 12 months persistence rates was assumed.

For FLS and no-FLS patients who initiated drug therapy, diagnostic and treatment costs include drug costs, bone mineral density (BMD) testing costs, general practice (GP) visit costs, and costs related to side effects. Annual drug costs, BMD testing and GP visit costs were retrieved from the National Development and Reform Commission of China (2018) [31]. It was assumed that subjects undergoing therapy had one GP visit per year and a dual-energy X-ray absorptiometry (DXA) per 2 years. In addition, considering serious adverse events (i.e., osteonecrosis of the jaw and atypical femoral fractures) associated with the use of bisphosphonate therapy are an increasing concern in the public media and for patients recently, which might cause extra costs; we therefore assumed that patients treated with alendronate required 0.041 more GP consultations during the first cycle (6 months) and 0.021 GP consultations during the following cycles of treatment, in line with a previous cost-effectiveness analysis [32]. Treatment costs stopped when patients discontinued therapies.

### FLS effects

Given the lack of treatment initiation and adherence data following FLS in China, we obtained relevant data from a literature review and meta-analysis [35]; these can be regarded as the average performance of any type of FLS. Therefore, according to the type of FLS-related data we obtained, we assumed the form of FLS in our study to be at the average level of intensity of intervention. Adherence refers to the extent to which a patient acts in accordance with the prescribed interval and dose of a dosing regimen [47]. Specifically, compared to no-FLS, the effect of FLS was included through three parameters. First, FLS are associated with costs. Estimates of the cost of FLS in mainland China were not available, and only one Taiwanese study [36] reported the FLS fee in their study; this was estimated to be US$133. In order to make the FLS cost in our study comparable to other previous studies (FLS coordinator or nurse practitioner-based care) [37, 38], for base-case analysis, a one-off FLS cost of US$200 were assumed. This cost was applied only to FLS attenders. Second, we assumed that 38% and 17.2% of patients initiated treatment in the FLS (attenders) and no-FLS group, respectively. Third, higher treatment adherence was assumed for FLS (attenders) in comparison with no-FLS (57% vs. 34.1%) [35].

With regard to FLS non-attenders, first, as we mentioned before, to make FLS and no-FLS branches comparable, each patient entered the model with the same baseline fracture risk, i.e., the same baseline fracture risk was assumed for FLS attenders and non-attenders; second, FLS non-attenders did not incur one-off FLS costs; third, given the lack of relevant research data for non-attenders, it was assumed that FLS non-attenders had the same treatment initiation (17.2%) and adherence (34.1%) rates as patients in the no-FLS pathway.

### Outcomes and analyses

For base-case analysis, at the FLS cost of US$200 for each patient, total healthcare costs and QALYs were estimated for both FLS and no-FLS pathways. The ICER was computed as the difference between FLS and no-FLS in terms of total costs (expressed in 2020 US dollars) divided by the difference in terms of QALYs. In addition, analyses were also conducted to determine the maximum FLS cost (per patient) that make the FLS cost-saving and cost-effective at the Chinese WTP threshold. The WTP threshold was set at US$10,500 per QALY gained, which was the one-time gross domestic product (GDP) per capita in China (year 2020) [48].

The one-way deterministic sensitivity analysis was conducted to assess the impact of a single parameter on the robustness of the model. A total of 1,000,000 trials were run for each analysis. The parameters were categorized into two types: FLS-related parameters and other parameters. For FLS-related parameters, ten one-way sensitivity analyses were conducted. First, considering that the cost of FLS in China is unclear, different costs (US$400 (doubled), US$600 (tripled)) were tested. Second, given the uncertainty of the effects of FLS on mortality, we did not include it in the base case; however, a lower mortality rate was assumed for FLS pathway in the sensitivity analysis (odds ratio (OR): 0.73), based on a previous meta-analysis [9]. Considering that a 27% reduction of mortality risk might be high, another one-way sensitivity analysis used a decrease of 20% (with OR = 0.876). Third, the FLS attendance rate was increased/decreased by 20%. Fourth, treatment adherence in the FLS pathway was also increased/decreased by 20%. Fifth, the proportion of patients initiating treatment in FLS pathway was halved and doubled separately. Of note, the increases/decreases mentioned above were in absolute percentages.

For other parameters, different values were assumed for the starting age, the proportion of women, the proportion of nursing home admissions, prevalence of osteoporosis, fracture costs, long-term costs, drug costs, treatment efficacy, baseline utility, and discount rate. A total of 23 one-way sensitivity analyses were conducted.

## Results

### Base-case and sensitivity analyses

Table 2 reports incremental costs and QALY and the ICER (expressed in cost per QALY gained) of FLS compared to no-FLS. For base-case analysis, in patients aged 65 years with a fracture, FLS was associated with lower lifetime total costs of US$501 in comparison with no-FLS but leads to 0.095 additional QALY gained, indicating that FLS was dominant (more QALY for less total costs) at a cost of US$200 per patient in the Chinese context. In addition, for every 100 patients (a mix of baseline fracture types) in the FLS, about four hip fractures, nine clinical vertebral fractures and three wrist fractures would be avoided. The maximum cost of FLS that makes the FLS to be cost-saving in the Chinese setting was US$958, and the maximum cost of FLS that makes the FLS to be cost-effective at the WTP threshold of US$10,500 per QALY gained was US$2495.

**Table 2:** Incremental cost, incremental QALY, and incremental cost-effectiveness ratio (cost (USD) per QALY gained) of FLS compared with no-FLS for patients with a recent fracture

	Incremental cost (FLS-no FLS)	Incremental QALY (FLS-no FLS)	ICER
Base case	− 501	0.095	Dominant
One-way sensitivity analyses (FLS-related parameters)			
FLS cost + 100%	− 367	0.097	Dominant
FLS cost + 200%	− 237	0.095	Dominant
Lower mortality rate for FLS pathway (OR = 0.73)	− 146	0.513	Dominant
Odds ratio of mortality + 20%	− 352	0.278	Dominant
FLS attendance rate − 20%	− 534	0.095	Dominant
FLS attendance rate + 20%	− 469	0.098	Dominant
Medication adherence in FLS − 20%	− 525	0.096	Dominant
Medication adherence in FLS + 20%	− 474	0.098	Dominant
One-way sensitivity analyses (other parameters)			
Age Starting age: 60	− 436	0.094	Dominant
Starting age: 70	− 161	0.041	Dominant
Starting age: 75	− 190	0.042	Dominant
Starting age: 80	196	0.012	16,451
Gender Proportion of women: 80%	− 677	0.119	Dominant
Proportion of women: 100%	− 815	0.136	Dominant
Proportion of women: 0%	− 72	0.040	Dominant
Proportion of patients entering nursing home + 100%	− 531	0.098	Dominant
Proportion of patients entering nursing home − 50%	− 489	0.100	Dominant
Osteoporosis prevalence + 20%	− 638	0.095	Dominant
+ 40%	− 771	0.106	Dominant
Nursing home cost − 50%	− 490	0.098	Dominant
Nursing home cost + 50%	− 525	0.096	Dominant
Fracture cost − 50%	− 175	0.098	Dominant
Fracture cost + 50%	− 831	0.097	Dominant
Drug cost − 50%	− 515	0.098	Dominant
Drug cost + 50%	− 477	0.096	Dominant
Baseline utility − 20%	− 507	0.077	Dominant
Baseline utility + 20%	− 500	0.116	Dominant
Treatment efficacy − 20%	− 507	0.096	Dominant
Treatment efficacy + 20%	− 507	0.099	Dominant
Discount rate: 3%	− 660	0.128	Dominant
Discount rate: 0%	− 1032	0.207	Dominant

*QALY* quality-adjusted life years, *FLS* fracture liaison service, *ICER* incremental cost-effectiveness ratio, *OR* odds ratio

For sensitivity analyses, our results were robust to numerous one-way sensitivity analyses overall. For FLS-related parameters, the FLS was still dominant even when the cost of FLS was tripled. In addition, the incremental cost and QALY were markedly affected by incorporating a lower mortality rate in the FLS pathway, where the QALY gained increased substantially if we assumed that FLS is associated with 27% reduction in the risk of mortality. No apparent impact on incremental cost and QALY were captured by varying the FLS attendance rate, medication adherence, or proportion of treatment initiation (neither when halved nor doubled).

For other parameters, the incremental cost and/or QALY were significantly affected by varying the starting age, the proportion of females, fracture costs, baseline utility, and discount rate. Specifically, it can be seen that FLS was associated with higher total costs with an incremental cost of US$196 and an additional 0.012 QALY gained for elderly patients (80 years and older). For these patients, the ICER was estimated at US$16,451 per QALY gained, so the FLS was not cost-effective at the Chinese WTP threshold. In addition, we found increasing the proportion of women led to more costs saved and QALYs gained, but if we included only male patients, the FLS was still dominant. Moreover, the incremental cost declined markedly compared to the base case by halving the costs of fracture. The incremental QALY varied largely by increasing/decreasing baseline utility value. A 3% discount rate was associated with higher incremental costs and QALYs gained. Our results remained robust (even more economic benefits) when adjusting the prevalence of osteoporosis to more accurately reflect the prevalence of osteoporosis in our target populations.

## Discussion

This study suggests that FLS dominated no-FLS (more QALYs, less costs) in patients aged 65 years with a recent fracture at a one-off FLS cost of US$200 per patient in the Chinese context. Our findings were robust to numerous one-way sensitivity analyses. For the FLS to be cost-saving and cost-effective at the Chinese WTP, the maximum cost of FLS was US$958 and US$2495, respectively. For elderly patients (80 years and older), the FLS was not cost-effective at the WTP threshold of US$10,500 per QALY gained. It can be explained that shorter life expectancy might render fewer opportunities for benefitting from the FLS.

An important implication of our study is that it seems potentially beneficial to implement FLS in China, given that it can prevent subsequent fractures and also lead to lifetime cost-savings. During the review process of our manuscript, a cost-effectiveness analysis of FLS in Taiwan was published [36]. Authors reported the benefits of FLS in patients with a hip fracture and concluded that post-fracture FLS care was cost-effective in comparison with usual care. In this study, the FLS cost was estimated to be US$133 per patient (a bit lower but still comparable to our assumption of US$200 per patient). If we apply their FLS cost, more favorable results were obtained (given the lower cost with a similar QALY). Of note, although results in the Taiwanese study were comparable to ours, there are many methodological differences. First, the Taiwanese study is a trial-based economic evaluation, which evaluated only the short-term benefit of FLS (2 years), while we performed a model-based economic evaluation to investigate the lifetime benefits of FLS. Second, the Taiwanese study used survival days as the effectiveness measurement and reported the net monetary benefit at a specific WTP, instead of using QALY as effectiveness and presenting the incremental cost-effectiveness ratio (as in our study). Third, the Taiwanese study presented the effect of FLS only on patients with a hip fracture, and only hip refractures were counted. However, our study assumed a mix of various fractures at baseline, and subsequent hip, vertebral, and wrist fractures were all taken into account.

Additionally, another Chinese study (which is the first reporting on FLS for vertebral fractures in China: patients aged 50 years or older with a recent vertebral compression fracture were recruited) [49] also reported that the dedicated fracture service seems a solution for preventing subsequent fractures as well as decreasing healthcare costs and concludes that the nationwide introduction of FLS in China is crucial. To ensure that patients with fractures are identified in a timely way and then invited to attend the FLS, building an FLS team with members from different fields of expertise, coordinated by a FLS coordinator, could be an alternative approach and a starting point for China. This could be similar to the FLS team in Taiwan [36], which consists of orthopedic physicians, spine surgeons, geriatricians, endocrinologists, rheumatologists, family physicians, and coordinators. A Canadian study [50] indicated that hiring an osteoporosis coordinator to identify patients with a fragility fracture and to coordinate their education, assessment, referral, and treatment of underlying osteoporosis could reduce subsequent fractures and lead to net hospital cost-savings. Moreover, the wide gap between fragility fractures and secondary prevention is a worldwide concern, especially in the Asia–Pacific region, where the IOF combined “Top Down” with “Bottom Up” activities across 18 countries, including China, in 2020–2021, with the goal of increasing by 50% the number of patients reached, by fostering FLS and improving quality of its services, as shown on the CTF International Map of Best Practice. This also shows that the establishment and development of FLS would be an effective approach for China.

However, it should be noted that two systematic reviews revealed significant heterogeneity in the form of FLS and huge variation in its effects [51, 52]. Wu et al. [52] summarized 57 FLS-related high-quality studies published up to February 2017 and identified that FLS varied considerably in terms of the key persons coordinating the FLS (physician, nurse, or other healthcare professional), setting (hospital, community), intensity (single, multiple), and duration (long or short term), which lead to further variation in clinical and economic benefits, and not all FLS could improve patient outcomes. This study also identified several components which contributed to FLS success, encompassing multidisciplinary involvement, being driven by a dedicated case manager, regular assessment and follow-up, multifaceted interventions, and patient education. In addition, the Best Practice Framework [53] and eleven patient-level key performance indicators [54] developed by the IOF could serve as guidelines for China in the design of adequate FLS and improving the quality of existing services. Only FLS with relatively high-quality and sufficient services will lead to clinical and economic benefits and have the potential to be cost-effective.

The economic impact and cost-effectiveness of FLS studies (worldwide) published up to 2016 were summarized in a previous systematic review [11]. In line with several cost-effectiveness analyses [38, 50, 55, 56], the FLS is a dominant (cost-saving) secondary fracture prevention strategy, compared to no-FLS or usual care. However, different assumptions were made in different studies, and our study has several strengths in comparison with other studies on the cost-effectiveness of FLS. First, the simulation model was adapted according to a previous Markov microsimulation model [12], which has been validated and applied in several prior studies [13, 32, 41, 57]. Second, in our model, patients in the FLS pathway distinguished attenders and non-attenders, which is in line with reality. This is an important differentiation, as the two groups might have different baseline fracture risk and treatment initiation rates, and the presence of FLS costs applied only to attenders. However, we found that no previous studies differentiate between FLS attenders and non-attenders and made similar assumptions; this might overestimate the lifetime costs and effects in the FLS pathway and affect ICER estimation. Third, the time-dependent persistence rate for oral bisphosphonates was assumed in our study, which is also revealed by real-world data [58] and applied in some cost-effectiveness analyses in osteoporosis [13, 34]; however, some studies on the cost-effectiveness of FLS [55, 56] just assumed a persistence rate of 100% or that the persistence rate remained the same for the whole duration of treatment. This is not realistic and might influence the result. Fourth, as we mentioned before, the effect of FLS on mortality is uncertain; therefore we did not include it in the base case. Although a lower mortality rate was assumed for FLS pathway in sensitivity analysis, we found no previous studies incorporated the effect of FLS on mortality. Fifth, the cost of side effects of oral bisphosphonate treatment was incorporated in our model; these costs were not included in most studies on the cost-effectiveness of FLS.

The main limitations of our study derive primarily from a lack of precision with several important parameters, such as the FLS attendance rate, excess mortality, and persistence with treatment. The estimates from other countries (most are from developed countries) were used, as there was no relevant data for China. However, considering the heterogeneity in healthcare systems between countries, the direct transferability of clinical and economic evidence might limit the accuracy of cost-effectiveness analysis; therefore, Chinese-specific real-world data is needed to confirm the results of our study and to better evaluate the cost-effectiveness of FLS in China. For future studies, we recommend collecting FLS-related real-world data, including the FLS attendance rate, FLS costs, initiation of treatment, and adherence in FLS. In addition, country-specific fracture-related data such as fracture incidence, excess mortality, baseline utility (for patients with a recent fracture) and fracture disutility, fracture costs, and medication adherence are also important. Second, given that no relevant data was available for FLS no-attenders, it was assumed that the probability of treatment initiation and the treatment adherence rate were the same as for patients in the no-FLS pathway and that FLS attenders and non-attenders had the same baseline fracture risk; these assumptions might not reflect the reality. Third, we assumed a mix of various fractures at baseline. The fracture type was not taken into account given the lack of relevant data (e.g., having osteoporosis and initiating medication according to the fracture type); therefore, we did not estimate the benefits of FLS per baseline fracture type, although the ICER estimation might depend on the baseline fracture type. Fourth, although a single utility of 0.7 was estimated based on the ICUROS study and assumed for patients with a recent fracture in our study, it might not represent the quality of life for different genders and age groups. Therefore, more detailed age- and gender-stratified baseline utilities should be applied to perform the estimation when relevant Chinese data are available. Fifth, a conservative assumption was made in our study that patients without osteoporosis did not initiate treatment. However, although according to some local guidelines (including Chinese guidelines), patients with grade 2 or 3 vertebral fractures should initiate treatment irrespective of their BMD status, we did not incorporate this in our model due to the lack of relevant data in China. We note that even if it were included, this would only lead to better economic benefit in FLS pathway. Sixth, when patients are discharged from hospital, most Chinese families prefer home care (entering a nursing home is not very common in China). The probability of entering a nursing home and costs of nursing home and home care remain uncertain in China. Therefore, expert opinion and data from previous studies were used. Seventh, as we mentioned before, we conservatively did not assume the extra increased risk when new fractures occurred during simulation, underestimating the benefits of FLS. Eighth, one similar study [38] assigned the disutility for side effects of oral bisphosphonate like dyspepsia and osteonecrosis of jaw; this was not incorporated in our model considering the uncertainty of the data. Ninth, the probabilistic sensitivity analysis was not conducted given the distributional data for most parameters are lacking; accordingly, the uncertainty in cost-effectiveness estimates could not be explored.

## Conclusion

FLS could potentially lead to lifetime cost-saving for patients who have experienced a fracture. Our study informs the potential cost-effectiveness of FLS and the knowledge gap in China; more future research incorporating Chinese-specific real-world data are needed to confirm the results of our study and to better evaluate the cost-effectiveness of FLS in China.

## Supplementary Information

Below is the link to the electronic supplementary material.

Supplementary file1 (DOCX 44 KB)

## Data Availability

All data analyzed as part of this study are included in this published article.
