# Supplementary material for: A model-based cost-effectiveness analysis of fracture liaison services in China
Source: Arch Osteoporos. 2022 Oct 5;17(1):132. doi: 10.1007/s11657-022-01170-1 (PMC9532296; doi:10.1007/s11657-022-01170-1)
Supplement: Supplementary file 1 — Supplementary file1 (DOCX 44 KB) [file 11657_2022_1170_MOESM1_ESM.docx]

**Electronic supplementary material**

**Appendix I: Osteoporosis-specific checklist** − specific items to include when reporting economic evaluations on osteoporosis

| **Item** | **Item no.** | **Recommendation** | **Reported on page no. / line no.** |
| --- | --- | --- | --- |
| Transition probabilities | 1 | Report the transition probabilities and how they were estimated (including increased fracture risk) | Method section + Table 1Subtitle: Osteoporosis prevalence, fracture risk and mortality |
| Excess mortality after fractures | 2 | Describe approaches and data sources used for the excess mortality after fractures | Method section + Table 1Subtitle: Osteoporosis prevalence, fracture risk and mortality |
| Fractures costs | 3 | Describe approaches and data sources used for fractures costs | Method section + Table 1 Subtitle: fracture cost |
| Fractures effects on utility | 4 | Describe approaches and data sources used for the effects of fractures on utility | Method section + Table 1 Subtitle: utility values |
| Treatment effect during treatment | 5 | Describe fully the methods used for the identification, selection, and synthesis of clinical effectiveness data (per fracture site) | Method section + Table 1 Subtitle: treatment effects; FLS effects |
| Treatment effect after discontinuation | 6 | Describe fully the methods used for the treatment effect after discontinuation | Method section Subtitle: treatment effects |
| Medication adherence | 7 | Describe approaches and data sources used for modeling medication adherence | Method section + Table 1 Subtitle: treatment effects; FLS effects |
| Treatment costs | 8 | Describe approaches and data sources used for therapy costs | Method section + Table 1 Subtitle: treatment effects; FLS effects |
| Treatment side effects | 9 | Describe approaches and data sources used for costs and utilities effects of adverse events | Method section + Table 1 Subtitle: treatment effects |

**Appendix II: CHEERS 2022 checklist—**Items to include when reporting economic evaluations of health interventions

| **Section/item** | **Item No.** | **Guidance for reporting** | **Reported in section** |
| --- | --- | --- | --- |
| **Title** | | | |
| Title | 1 | Identify the study as an economic evaluation and specify the interventions being compared. | Title section |
| **Abstract** | | | |
| Abstract | 2 | Provide a structured summary that highlights context, key methods, results, and alternative analyses. | Abstract section |
| **Introduction** | | | |
| Background and objectives | 3 | Give the context for the study, the study question, and its practical relevance for decision making in policy or practice. | Introduction section |
| **Methods** | | | |
| Health economic analysis plan | 4 | Indicate whether a health economic analysis plan was developed and where available. | Method section |
| Study population | 5 | Describe characteristics of the study population (such as age range, demographics, socioeconomic, or clinical characteristics). | Method section  Subtitle: Model structure |
| Setting and location | 6 | Provide relevant contextual information that may influence findings. | Introduction and method section |
| Comparators | 7 | Describe the interventions or strategies being compared and why chosen. | Method section |
| Perspective | 8 | State the perspective(s) adopted by the study and why chosen. | Method section |
| Time horizon | 9 | State the time horizon for the study and why appropriate. | Method section |
| Discount rate | 10 | Report the discount rate(s) and reason chosen. | Method section  Subtitle: Model structure |
| Selection of outcomes | 11 | Describe what outcomes were used as the measure(s) of benefit(s) and harm(s). | Method section  Subtitle: Model structure |
| Measurement of outcomes | 12 | Describe how outcomes used to capture benefit(s) and harm(s) were measured. | Method section  Subtitle: Model structure Outcomes and analyses |
| Valuation of outcomes | 13 | Describe the population and methods used to measure and value outcomes. | Method section  Subtitle: Model structure  Outcomes and analyses |
| Measurement and valuation of resources and costs | 14 | Describe how costs were valued. | Method section  Subtitle: Fracture cost  Treatment effects |
| Currency, price date, and conversion | 15 | Report the dates of the estimated resource quantities and unit costs, plus the currency and year of conversion. | Method section  Subtitle: Fracture cost |
| Rationale and description of model | 16 | If modeling is used, describe in detail and why used. Report if the model is publicly available and where it can be accessed. | Introduction and method section |
| Analytics and assumptions | 17 | Describe any methods for analysing or statistically transforming data, any extrapolation methods, and approaches for validating any model used. | Method section  Subtitle: Treatment pathways Osteoporosis prevalence, fracture risk and mortalityUtility valuesTreatment effectsFLS effects |
| Characterising heterogeneity | 18 | Describe any methods used for estimating how the results of the study vary for subgroups. | Method section  Subtitle: Outcomes and analyses |
| Characterising distributional effects | 19 | Describe how impacts are distributed across different individuals or adjustments made to reflect priority populations. | NA |
| Characterising uncertainty | 20 | Describe methods to characterise any sources of uncertainty in the analysis. | Method section  Subtitle: Outcomes and analyses |
| Approach to engagement with patients and others affected by the study | 21 | Describe any approaches to engage patients or service recipients, the general public, communities, or stakeholders (such as clinicians or payers) in the design of the study. | NA |
| **Results** | | | |
| Study parameters | 22 | Report all analytic inputs (such as values, ranges, references) including uncertainty or distributional assumptions. | Method section and Table 1 |
| Summary of main results | 23 | Report the mean values for the main categories of costs and outcomes of interest and summarise them in the most appropriate overall measure. | Results section and Table 2 |
| Effect of uncertainty | 24 | Describe how uncertainty about analytic judgments, inputs, or projections affect findings. Report the effect of choice of discount rate and time horizon, if applicable. | Results section and Table 2 |
| Effect of engagement with patients and others affected by the study | 25 | Report on any difference patient/service recipient, general public, community, or stakeholder involvement made to the approach or findings of the study | NA |
| **Discussion** | | | |
| Study findings, limitations, generalizability, and current knowledge | 26 | Report key findings, limitations, ethical or equity considerations not captured, and how these could affect patients, policy, or practice. | Discussion section |
| **Other relevant information** | | | |
| Source of funding | 27 | Describe how the study was funded and any role of the funder in the identification, design, conduct, and reporting of the analysis | Source of funding section |
| Conflicts of interest | 28 | Report authors conflicts of interest according to journal or International Committee of Medical Journal Editors requirements. | Conflicts of interest section |

NA not applicable
